# Supplementary material for: Effect of distributing locally produced cloth facemasks on COVID-19-like illness and all-cause mortality–a cluster-randomised controlled trial in urban Guinea-Bissau
Source: PLOS Glob Public Health. 2024 Feb 13;4(2):e0002901. doi: 10.1371/journal.pgph.0002901 (PMC10863890; doi:10.1371/journal.pgph.0002901)
Supplement: S1 Text — (DOCX) [file pgph.0002901.s001.docx]

**Supplementary Materials**

Effect of distributing locally produced cloth facemasks on COVID-19-like illness and all-cause mortality – a Cluster-Randomised Controlled Trial in urban Guinea-Bissau

Line M Nanque, Andreas M Jensen, Arthur Diness, Sebastian Nielsen, Carlos Cabral, Dylan Cawthorne, Justiniano S D Martins, Elsi J C Ca, Kjeld Jensen, Cesario L Martins, Amabelia Rodrigues, Ane B Fisker

[Supplementary Methods 2](#_Toc153957704)

[Setting 2](#_Toc153957705)

[Study design and randomisation 2](#_Toc153957706)

[Intervention 2](#_Toc153957707)

[Assessment of outcomes 5](#_Toc153957708)

[Telephone follow-up 5](#_Toc153957709)

[Direct observations 5](#_Toc153957710)

[End of trial visit 5](#_Toc153957711)

[Statistical analyses 6](#_Toc153957712)

[Primary analysis 6](#_Toc153957713)

[Direct observations 6](#_Toc153957714)

[Supplementary Results 8](#_Toc153957715)

[Enrolment, baseline characteristics and completeness of follow-up 8](#_Toc153957716)

[Direct observations 10](#_Toc153957717)

[Effects of facemask distribution 11](#_Toc153957718)

[Pretrial mortality 18](#_Toc153957719)

[References 19](#_Toc153957720)

# **Supplementary Methods**

## **Setting**

Since 2002, the Bandim Health Project covers six districts with a total of 37 zones divided into 324 clusters (Fig A). Each cluster has a median of 232 individuals, interquartile range (IQR): 194-261.


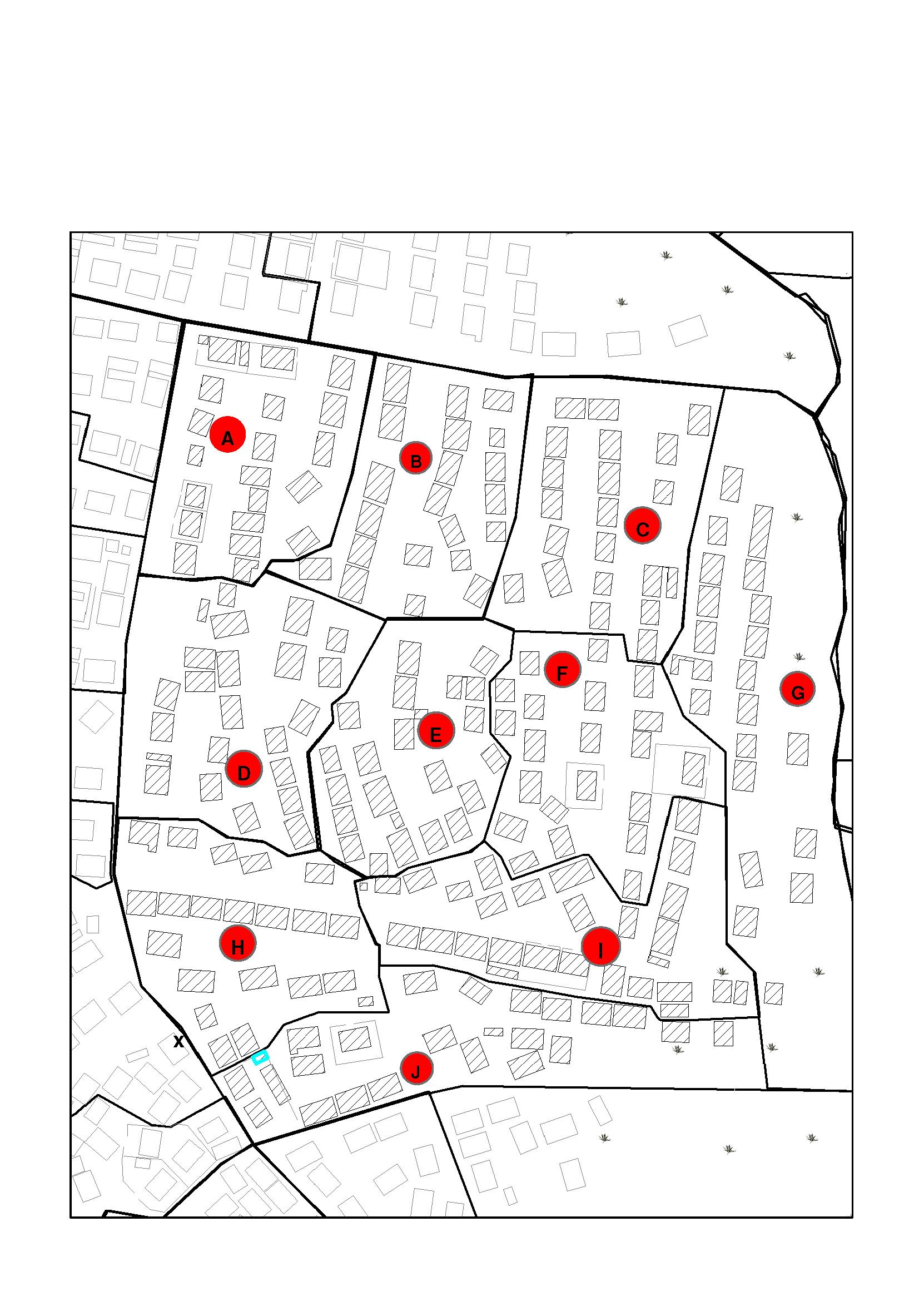


**Fig A: Example of a Bandim Health Project are study zone with clusters A-J.**

## **Study design and randomisation**

All clusters were classified according to size of the target population, age distribution (proportion of individuals over 50) and socioeconomic status (proportion of households with functioning electricity during the past 2 years). Clusters were randomized to intervention or control arm. To ensure balancing, 10,000 random combinations of cluster allocations were drawn per district. We independently selected a random combination per district, replacing the selection if a combination with imbalances between the number of participants, age distribution above 50 years of age and proportion of individuals from households with functioning electricity was selected. Randomisation was performed by a study team member not involved in the fieldwork (SN). Further details can be found in the analysis plan available at <https://clinicaltrials.gov/ProvidedDocs/66/NCT04471766/SAP_000.pdf>.

## **Intervention**

A cloth facemask was developed by volunteers at Engineers Without Borders Denmark. The developed mask had to be made from locally available materials, be producible by local tailors, and provide protection against the airborne droplets that transmit COVID-19, yet still be breathable. Different fabric types were inspected and tested for breathability and a commonly available plain-weave cotton was selected (Fig B). The design specifically aimed to ensure a good fit around the face.


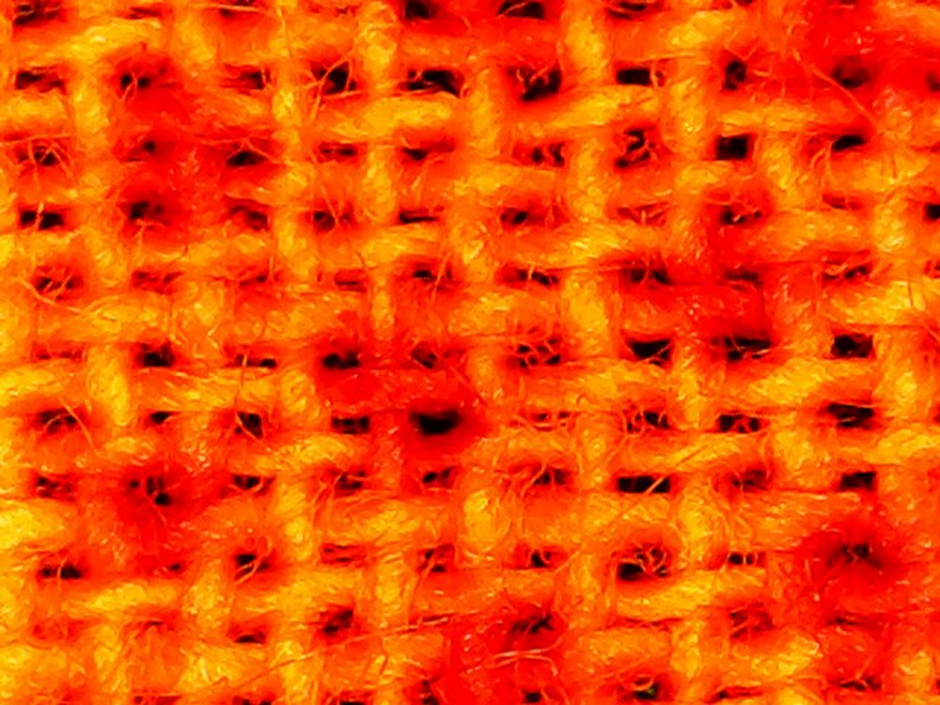


**Fig B: Magnification (20X) of a single layer of locally available plain-weave cotton fabric after several wash-dry cycles** (Photographed by: Dylan Cawthorne)

This resulted in a facemask design with a thin metal wire at the nose bridge and two elastic straps that went around the back of the head and neck, rather than just around the ears, to hold the mask in place during movement. The shape of the mask was developed such that it fit the face closely, but still had enough room at the front, so it did not interfere with speaking. Several iterations of prototype fabric masks were produced, along with a flat pattern for cutting the panels; it was determined that one size mask would not fit everyone, so the pattern included small, medium, large, and extra-large sizes. A detailed training video for tailors on how to cut and sew the mask was produced (<https://youtu.be/Rl_ph5eCazY>), and a series of video conference calls between BHP, IUG and the tailors took place.

Quality was ensured through visual inspection of photos and videos, during the early production phase. During later phases, study supervisors inspected delivered masks before they were washed, ironed and prepared for distribution.

A sample of the cloth facemasks produced by investigators using washed plain weaved cotton fabric bought in Guinea-Bissau, were tested by the company FORCE Technology according to EN 149 for medical facemasks[1]. The tested flow rates were 30 and 150 l/min to simulate in- and exhalation, and the particles tested were paraffin oil (0.6 µm) and NaCl (0.4µm). Thus, the filtration ability was tested for sizes substantially smaller than the droplet sizes in e.g. a sneeze (3µm)[2]. Using this standard, FFP2 masks should filter at least 94% of the particles. The results indicated an 11-19% filtration rate dependent on particle size. Airflow resistance was not measured. Testing was not repeated for the masks which were subsequently produced, and acceptability was not investigated.

Intervention group participants were provided with masks in size small, medium, large or extra-large dependent on the field worker’s visual assessment of suitable size. Participants in both the intervention and control group were provided with advice on prevention COVID-19 transmission (Fig C).


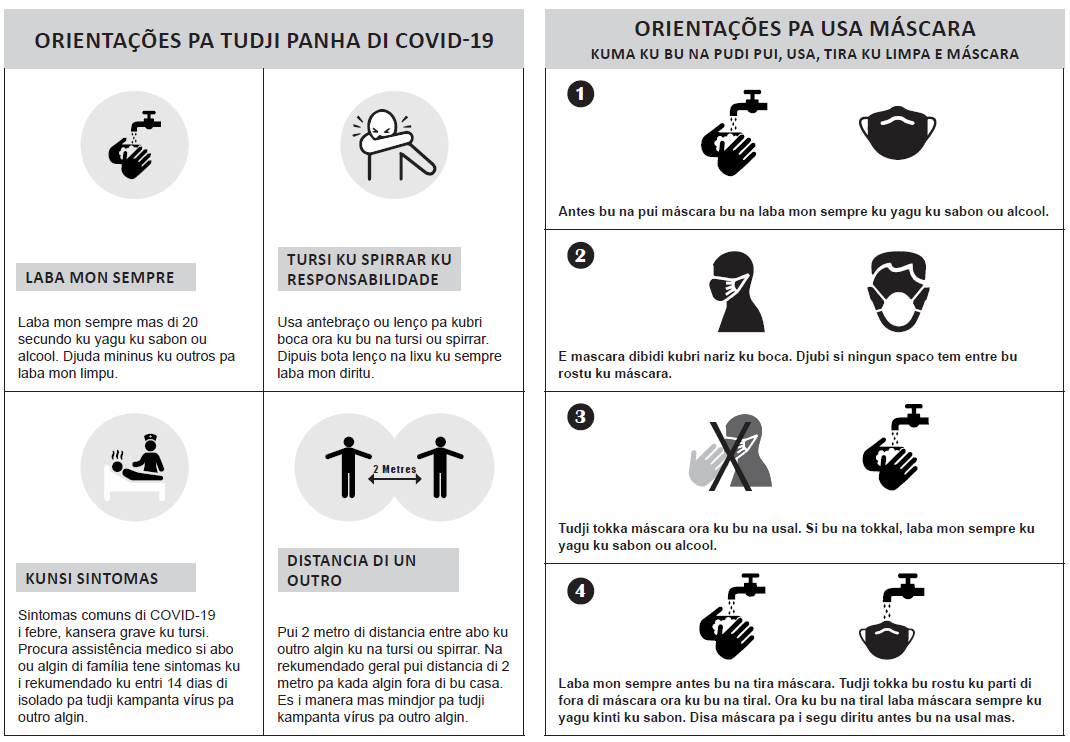


**Fig C: Pictogram from information sheet distributed to intervention and control group.**

Note: Information sheet prepared by Engineers without Boarders and translated to Portuguese Creole.

**
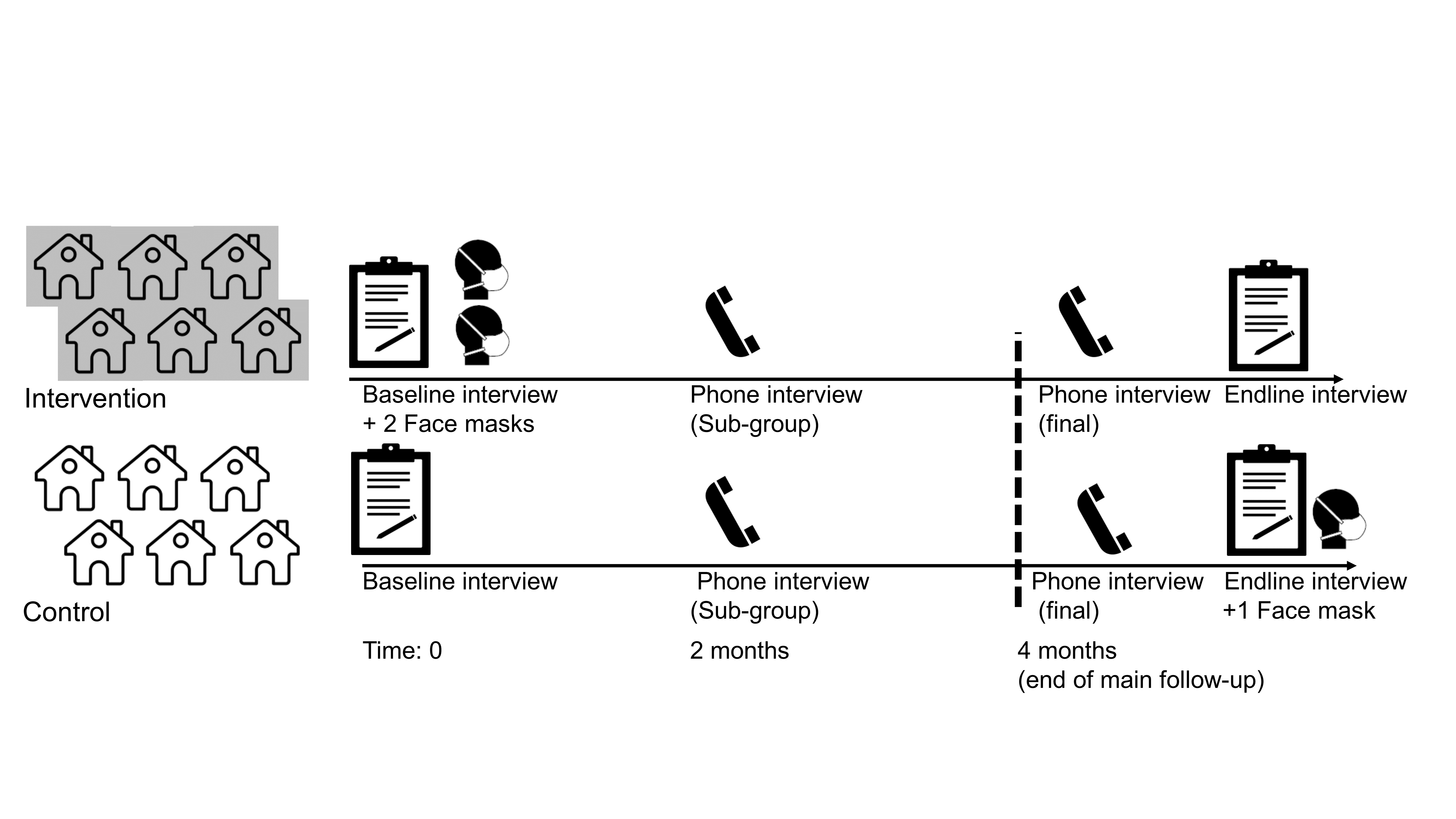
**

**Fig D: Trial design Guinea-Bissau Facemask trial.**

Note: at telephone interviews information on mask use, exposure to large groups of people and symptoms and care seeking behaviour during episodes of illness since enrolment/prior call was collected. Dates of illness episodes were not collected but dates of consultation were. At both telephone interviews and home visit with interviews information on vital status (and where relevant date of death) was collected. Icons from “The Noun Project”, Wikimedia Commons (<https://commons.wikimedia.org/>. Mask icon drawn by co-author Dylan Cawthorne)

## **Assessment of outcomes**

The four prespecified primary outcomes were registered at clinicaltrials.gov and the analyses are described in the statistical analysis plan. However, consultations for COVID-19-like illness is not clearly described as a separate outcome in the original protocol.

### **Telephone follow-up**

Due to curfews and other logistical challenges, working hours during the pandemic were limited. We prioritised mask production, quality control and distribution and therefore encountered delays in conducting the telephone follow-up. Hence, we were only able to implement more frequent calls in a subset of the population enrolled in Cuntum-II and Bandim-I, zone 8. Here we implemented two telephone follow-up interviews: the 1st interview around 2 months of follow-up and the 2nd interview after 4 months of follow-up. In the remaining clusters, calls were only made after 4 months (Fig D).

### **Direct observations**

During the full study period, we conducted direct observations of use of facial masks. Observations were conducted outside, from a distance, in all clusters on Monday-Friday from 8am-3pm. The observer spent approximately 2 hours per “observation spot” in a cluster to register the number of people in sight and for everyone whether they wore a mask, whether the mask covered nose and mouth and whether the mask worn was the study cloth facemask. The study cloth facemasks were recognisable as they had two elastic band extending across the back of the head and the neck, and covered the entire jaw, including the area between the jaw-angle and the ear (Fig E).

### **End of trial visit**

In addition to the end of trial visits, we are conducting verbal autopsies for all deaths of participants registered during the study period to assess whether the overall effect of distributing facemasks differ by cause of death based on classifications as likely COVID-19, other infectious, non-infectious. However, due to limited resources the verbal autopsies are not yet finalised and will be reported elsewhere.


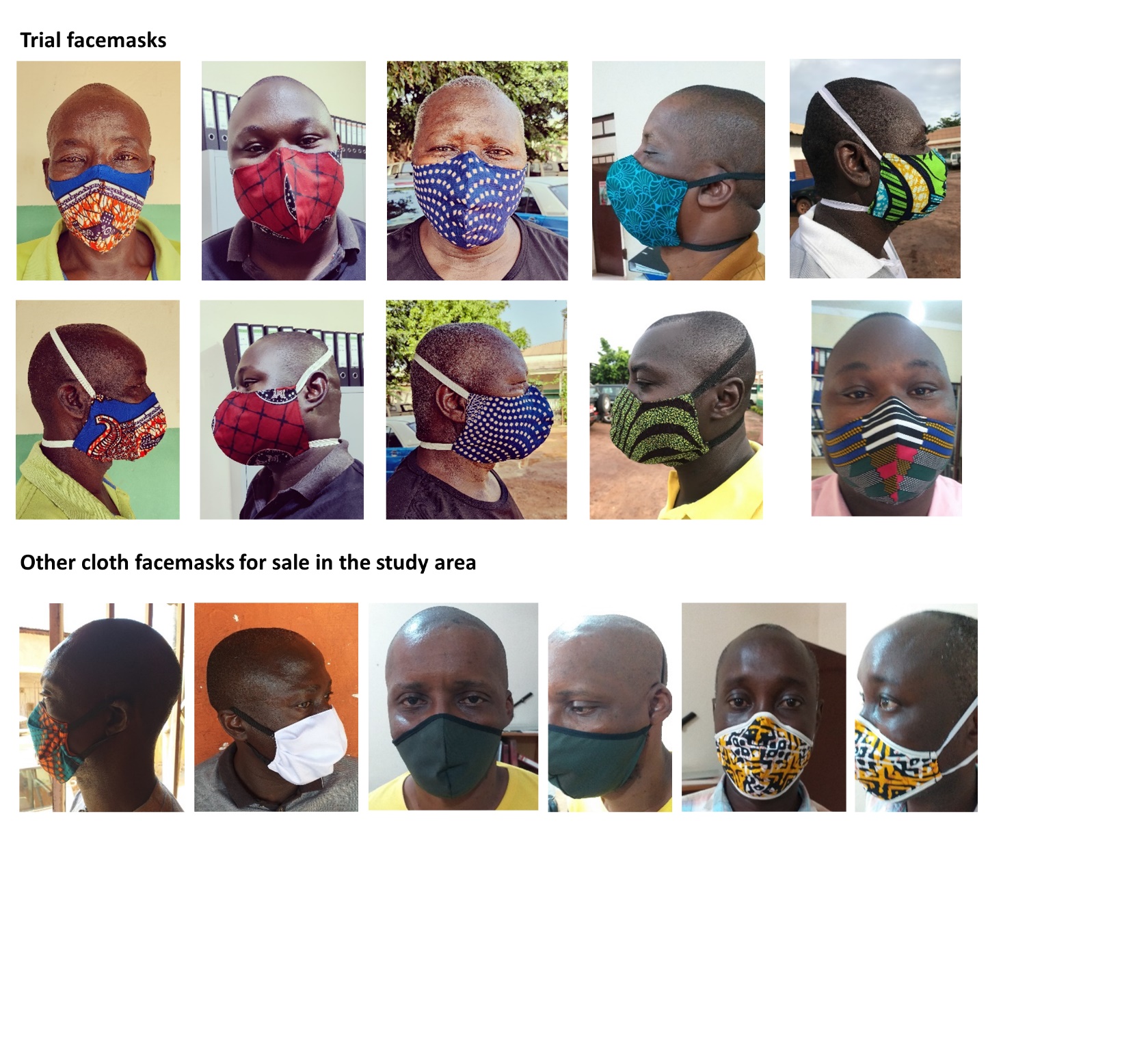


**Fig E: Trial facemasks and other facemasks worn by BHP staff.**

## **Statistical analyses**

### **Primary analysis**

The primary analysis included all individuals for whom a telephone interview was conducted. For the balancing variables, we expressed the variables as the proportional departure from the mean (distance to mean divided by the mean). We used cluster rather than zone as the cluster variable for the generalised estimating equation (GEE)-based correction for cluster, as the computation of the estimates were hampered. All statistical tests were 2-tailed with p≤0.05 considered statistically significant.

### **Direct observations**

We compared the proportion of people observed wearing masks, observed with masks covering nose and mouth and observed with study masks in linear regression models with the number of people observed per session as the weights. Unlike the analyses of the morbidity and mortality outcomes, we had not specified these analyses in the analysis plan.


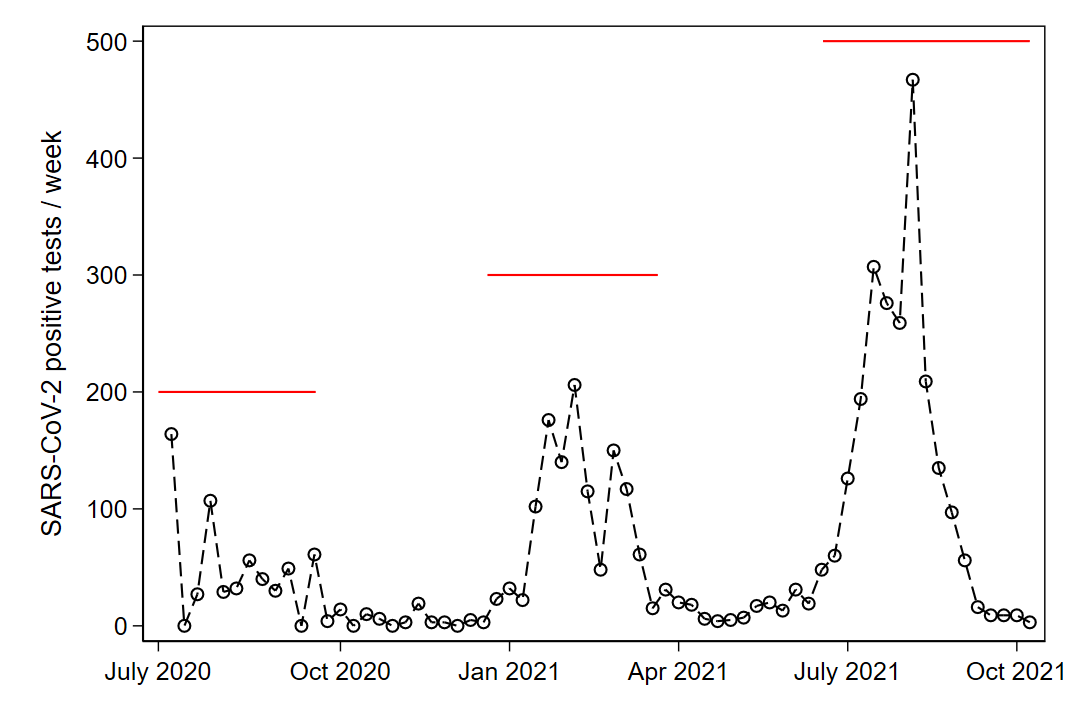


**Fig F: SARS-CoV-2 positive test in Guinea-Bissau. Red horizontal lines indicate the three time periods classified as higher risk of transmission.**

Note: Data from Our World in Data (<https://ourworldindata.org/coronavirus/country/guinea-bissau> downloaded on 03 April 2022.

# **Supplementary Results**

### **Enrolment, baseline characteristics and completeness of follow-up**

Among the 38,197 persons identified as potentially eligible in the HDSS database, 2049 were not eligible to be enrolled when visited by the enrolment teams. Among them 114 had died, 65 in the intervention arm and 49 in the control arm. Thus, the proportion of pretrial deaths tended to be higher before enrolment (crude relative risk = 1.35, 95%CI: 0.93-1.95) in the intervention clusters than in the control clusters.

During the enrolment visits, 2126 new residents were registered in the intervention arm and 1447 in the control arm. Thus, people in the intervention group were 44%, 95%CI: 35-54%, more likely to be new registrations than in the control arm.


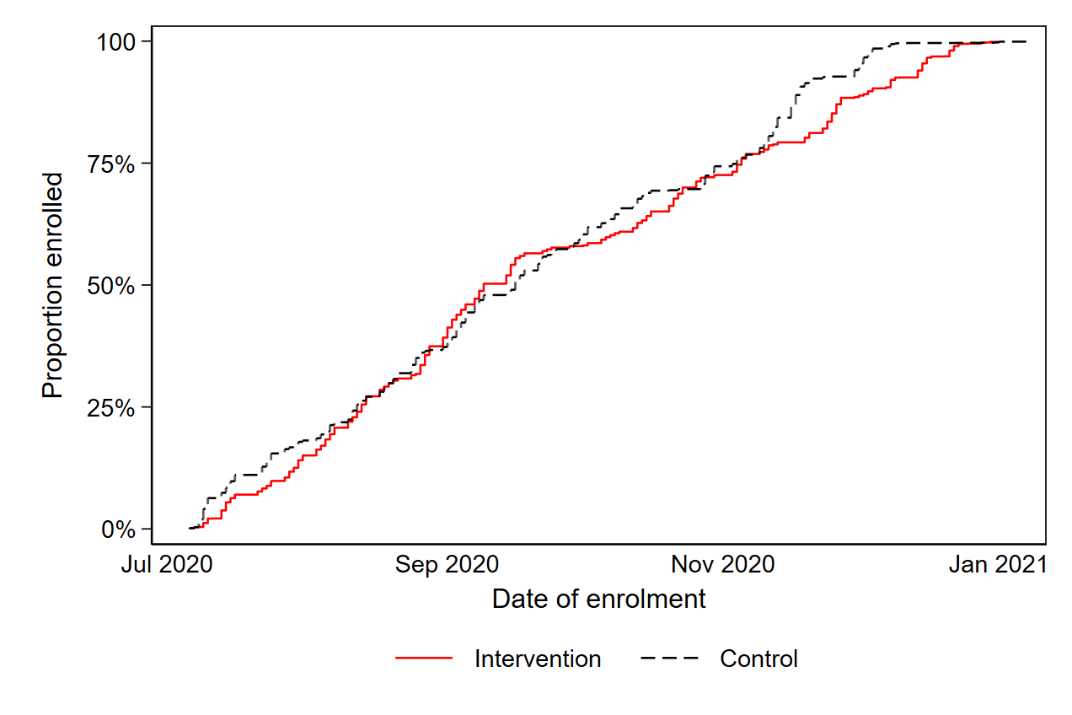


**Fig G: Enrolment rate by trial arm.**

**Table A**: **Summary of follow-up information by intervention and control group**

|  | **Intervention  [n, (%)]** | **Control  [n, (%)]** | **P value, test of same distribution** |
| --- | --- | --- | --- |
| Eligible for telephone interviews | 19,969 | 19,605 |  |
| Total number of calls | 31,163 | 29,514 |  |
| Number of successful calls (% of calls) | 20,899 (67) | 20,118 (68) | <0.001 |
| Number of participants providing information him-/herself^1^ | 8017 (39) | 7254 (36) | <0.001 |
| Number of participants with successful call (% of eligible for calls) | 16,779 (84) | 16,527 (84) | 0.45 |
| **Among participant with successful call** |  |  |  |
| Number of participants with >1 follow follow-up telephone call | 3879 (23) | 3370 (20) | <0.001 |
| Time between enrolment and first successful telephone call, median (IQR) | 210 (99-241) | 231 (150-250) | <0.001 |
| **Exposure to large groups of people** |  |  |  |
| Reported to have attended events with >20 persons not living with participant^2^ | 15,133 (95) | 14,989 (96) | 0.01 |
| Frequently attended event (School/work/market)^3^ | 13,533 (90) | 13,588 (91) | <0.001 |
| Reported to have been in contact with a person with COVID-19^4^ | 176 (1) | 154 (1) | 0.30 |
| **Mask compliance**^5^ |  |  | 0.11 |
| Never use | 132 (1) | 160 (1) |  |
| Sometimes | 29 (0) | 30 (0) |  |
| When leaving home | 14067 (92) | 13059 (92) |  |
| Often | 1013 (7) | 903 (6) |  |
| **Type of mask used**^6^ |  |  | <0.001 |
| Study mask | 8587 (59) | 574 (5) |  |
| Medical mask | 5453 (37) | 7315 (67) |  |
| Cloth mask of other origin | 635 (4) | 3020 (28) |  |
| Medical/Cloth | 3 (0) | 12 (0) |  |

1: Missing information on informant for 230 interviews (intervention: 122, control: 108); 2: Missing information on exposure to large groups for 1756 persons (intervention: 879, control: 877); 3: Among those with exposure to large groups. Missing information on frequency of exposure for 112 persons (intervention: 63, control: 49); 4: Missing information on exposure to large groups for 3051 persons (intervention: 1502, control: 1549); 5: Missing information on mask use for 3913 persons (intervention: 1538, control: 2375); 6: Missing information on mask use for 7707 persons (intervention: 2101, control: 5606)

### **Direct observations**

We conducted 394 observation sessions (intervention clusters: 189; control: 205) between 14 October 2020 and 17 June 2021. A session lasted 2 hours (median: 123 minutes, IQR: 121-126 minutes) and the median number of persons observed per session was 443, IQR: 368-523 with little difference between the intervention areas (438, IQR: 368-508) and control areas (443, IQR: 373-537). A total of 177,950 observations of mask use were made (intervention: 84,799; control: 93,151 persons).

The proportion wearing a mask differed over time, with less than half wearing a mask before January 22, 2021, followed by a sharp increase and a gradual decline (Fig 3).

Overall, the proportions observed wearing masks, did not differ significantly between the observations made in the intervention (41%) and control clusters (37%) (p=0.07). The numbers were similar when restricting the observation prior to before 23 May 2021 (4 months after the end of enrolment): intervention clusters: 44% control clusters: 40%. The difference did not change when adjusting for period and the linear decline after January 22, 2021 (Difference in mask use: 3%, 95%CI: 0-6%).

Masks were far from always worn correctly. For only 19% of the people observed from an address in the intervention area did the person wear a mask covering nose and mouth, while this was 17% among people observed from a control group address (Fig H).


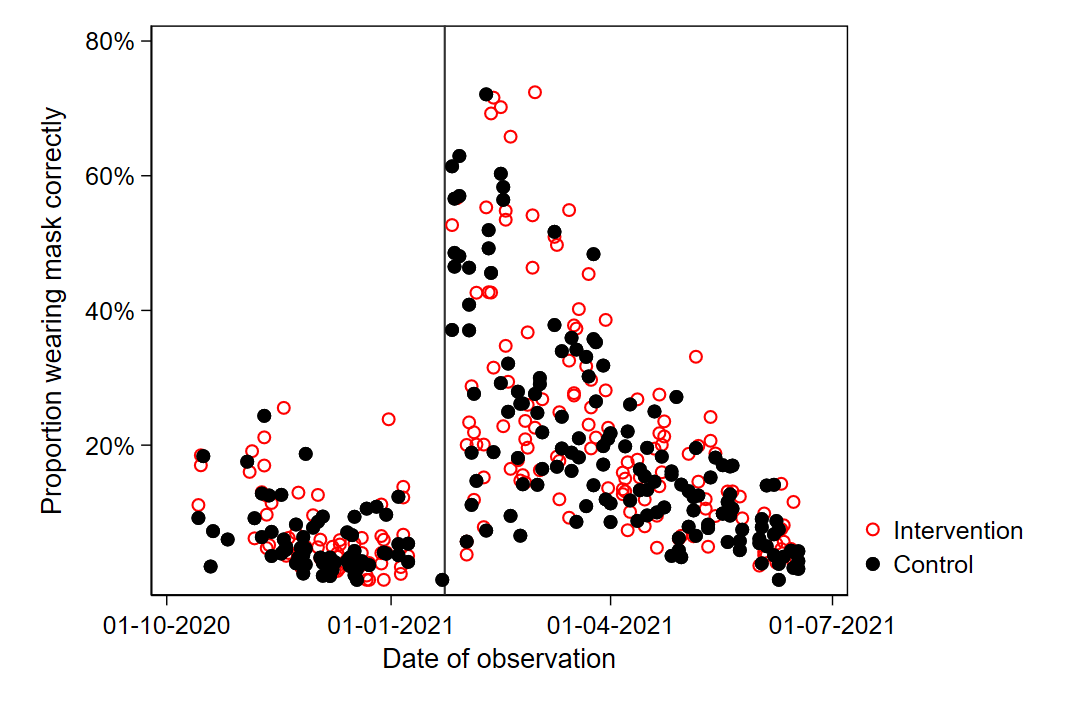


**Fig H: Direct observations. Proportion of individuals observed wearing a face mask correctly (covering both mouth and nose) during each 2-hour observation session.**

Note: Vertical line indicates January 22, 2021, when a sudden change in observed facemask use occurred.

Finally, the masks classified as study masks were only worn by a small proportion overall: by 3.1% of people observed in the intervention groups and by 2.3% of the people observed from an observation post at a control group address (Fig I).


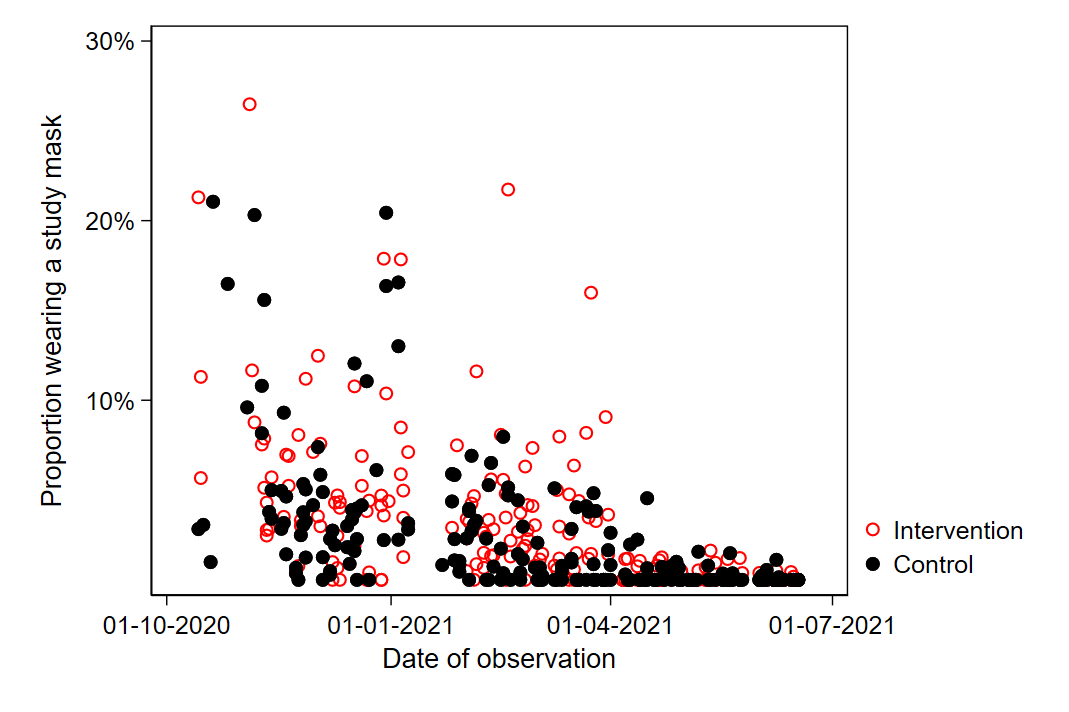


**Fig I: Direct observations. Proportion of individuals observed wearing a study face mask during each 2-hour observation session.**

### **Effects of facemask distribution**

Among the 11 admissions in participants who also reported COVID-19-like illness, only one (intervention) was reported to have been diagnosed as COVID-19; 6 with malaria, 1 pneumonia, 1 tuberculosis (both COVID-19 test negative), 1 dehydration and 1 heart problem.

**Table B: Effects of facemask distribution on the primary and secondary outcomes, stratified by potential effect modifiers.**

|  | **Self-reported COVID-19-like illness** | | | | **Consultations for COVID-19-like illness** | | | | **All-cause mortality** | | | |
| --- | --- | --- | --- | --- | --- | --- | --- | --- | --- | --- | --- | --- |
|  | **Interv. [% (events/n)]** | **Cont. [% (events/n)]** | **OR (95%CI)^a^** | **P-value^b^** | **Interv. [% (events/n)]** | **Cont. [% (events/n)]** | **OR (95%CI)^a^** | **P-value^b^** | **Interv. [% (events/n)]** | **Cont. [% (events/n)]** | **OR (95%CI)^a^** | **P-value^a^** |
| **Age in decades** | | | | | | | | | | | | |
| 10-19 | 0.75 (35/4664) | 0.85 (39/4574) | 0.88 (0.49-1.60) | 0.16**^c^** | 0.34 (15/4462) | 0.21 (9/4364) | 1.58 (0.58-4.29) | 0.12**^c^** | 0.07 (4/5599) | 0.00 (0/5479) | NA | 0.27^c^ |
| 20-29 | 1.53 (73/4781) | 1.65 (77/4680) | 0.90 (0.60-1.36) |  | 0.51 (23/4518) | 0.72 (32/4430) | 0.69 (0.40-1.17) |  | 0.09 (5/5640) | 0.02 (1/5490) | 4.70 (0.56-39.52) |  |
| 30-39 | 1.75 (62/3533) | 2.51 (86/3420) | 0.68 (0.44-1.05) |  | 0.48 (16/3360) | 1.04 (34/3261) | 0.46 (0.26-0.82) |  | 0.21 (9/4211) | 0.15 (6/4087) | 1.41 (0.50-3.94) |  |
| 40-49 | 2.07 (43/2078) | 2.24 (46/2050) | 0.91 (0.53-1.57) |  | 0.84 (17/2014) | 0.76 (15/1986) | 1.11 (0.51-2.41) |  | 0.32 (8/2507) | 0.24 (6/2467) | 1.22 (0.43-3.46) |  |
| 50-59 | 1.70 (16/942) | 2.03 (21/1032) | 0.84 (0.39-1.81) |  | 0.44 (4/915) | 0.10 (1/994) | 4.52 (0.51-40.03) |  | 0.64 (7/1091) | 0.51 (6/1187) | 1.21 (0.39-3.71) |  |
| 60-69 | 1.69 (9/533) | 2.00 (11/549) | 0.79 (0.27-2.32) |  | 0.97 (5/517) | 0.76 (4/527) | 1.31 (0.26-6.54) |  | 0.95 (6/632) | 1.43 (9/628) | 0.64 (0.23-1.82) |  |
| 70-79 | 2.42 (6/248) | 3.62 (8/221) | 0.68 (0.18-2.51) |  | 0.90 (2/223) | 0.00 (0/201) | NA |  | 4.56 (13/285) | 3.42 (9/263) | 1.29 (0.50-3.33) |  |
| **Reported to have attended event with >20 people not from own household** | | | | | | | | | | | | |
| No | 1.69 (13/767) | 3.48 (23/661) | 0.50 (0.23-1.08) | 0.95 | 0.53 (4/757) | 1.07 (7/652) | 0.50 (0.16-1.55) | 0.36 |  |  |  |  |
| Yes | 1.52 (230/15133) | 1.77 (265/14989) | 0.85 (0.60-1.21) |  | 0.51 (77/15057) | 0.59 (88/14939) | 0.86 (0.57-1.29) |  |  |  |  |  |
| **Frequency of attending event** | | | | | | | | | | | | |
| Occasionally | 1.23 (19/1548) | 1.77 (24/1358) | 0.74 (0.41-1.34) | 0.16 | 0.45 (7/1543) | 0.59 (8/1354) | 0.84 (0.33-2.16) | 0.99 |  |  |  |  |
| Frequently | 1.56 (212/13571) | 1.78 (242/13617) | 0.87 (0.60-1.26) |  | 0.52 (70/13536) | 0.59 (80/13597) | 0.84 (0.54-1.31) |  |  |  |  |  |
| **Reported to have been in contact with a person with COVID-19 (Y/N)** | | | | | | | | | | | | |
| No | 1.52 (229/15101) | 1.83 (272/14824) | 0.81 (0.58-1.14) | 0.37 | 0.52 (78/15072) | 0.61 (91/14803) | 0.84 (0.56-1.27) | 0.79 |  |  |  |  |
| Yes | 4.55 (8/176) | 6.49 (10/154) | 0.51 (0.19-1.41) |  | 1.71 (3/175) | 1.97 (3/152) | 0.67 (0.13-3.47) |  |  |  |  |  |
| **Ceiling in household** | | | | | | | | | | | | |
| No | 1.84 (109/5940) | 2.45 (144/5876) | 0.72 (0.48-1.08) | 0.25 | 0.69 (39/5657) | 0.93 (52/5613) | 0.81 (0.49-1.33) | 0.64 | 0.26 (18/6943) | 0.22 (15/6739) | 1.32 (0.65-2.71) | 0.98 |
| Yes | 1.25 (129/10358) | 1.33 (136/10245) | 0.92 (0.61-1.40) |  | 0.41 (41/9895) | 0.41 (40/9766) | 0.97 (0.54-1.73) |  | 0.26 (32/12397) | 0.18 (22/12371) | 1.33 (0.77-2.32) |  |
| **Number of enrolled people sleeping in same room** | | | | | | | | | | | | |
| 1 | 1.81 (61/3375) | 2.15 (77/3588) | 0.80 (0.53-1.22) | 0.77 | 0.47 (15/3209) | 1.05 (36/3413) | 0.46 (0.25-0.85) | 0.02 | 0.35 (14/4053) | 0.28 (12/4279) | 1.24 (0.58-2.65) | 0.70 |
| 2-3 | 1.52 (140/9232) | 1.87 (172/9213) | 0.76 (0.52-1.11) |  | 0.57 (50/8834) | 0.54 (48/8839) | 0.99 (0.59-1.66) |  | 0.25 (27/10886) | 0.16 (18/10918) | 1.50 (0.85-2.64) |  |
| 4+ | 1.06 (30/2841) | 1.04 (29/2776) | 1.05 (0.56-1.99) |  | 0.48 (13/2727) | 0.27 (7/2633) | 1.77 (0.66-4.76) |  | 0.18 (6/3357) | 0.16 (5/3225) | 1.21 (0.36-4.05) |  |
| **Number of children <10 in household at enrolment** | | | | | | | | | | | | |
| 0 | 1.60 (74/4622) | 2.03 (90/4427) | 0.62 (0.39-1.00) | 0.08 | 0.50 (22/4388) | 0.60 (25/4185) | 0.62 (0.36-1.08) | 0.45 | 0.24 (14/5794) | 0.16 (9/5500) | 1.42 (0.59-3.44) | 0.54 |
| 1 | 1.50 (71/4723) | 1.37 (61/4445) | 1.01 (0.61-1.69) |  | 0.42 (19/4506) | 0.47 (20/4275) | 0.84 (0.43-1.66) |  | 0.34 (19/5552) | 0.15 (8/5292) | 2.11 (0.92-4.87) |  |
| 2-3 | 1.36 (74/5451) | 1.43 (81/5667) | 1.01 (0.66-1.53) |  | 0.60 (31/5205) | 0.46 (25/5413) | 1.39 (0.70-2.77) |  | 0.20 (13/6455) | 0.27 (18/6585) | 0.74 (0.35-1.55) |  |
| 4+ | 1.26 (25/1983) | 2.82 (56/1988) | 0.56 (0.28-1.10) |  | 0.52 (10/1910) | 1.32 (25/1891) | 0.47 (0.16-1.34) |  | 0.28 (6/2164) | 0.09 (2/2225) | 3.19 (0.61-16.59) |  |

a: Estimated in logistic regression models with generalised estimating equation-based correction for cluster. Adjusted for zone and the cluster balancing variables: number of people, proportion aged >50 years and proportion of households with functioning electricity. b: P-value: Test of same effect across strata. c: Interaction tested in a model where the two youngest age groups (10-19 and 20-29) were with combined

**Table C: Effects of facemask distribution on health centre consultations and mortality, extended follow up.**

|  |  | **Intervention   [% (events/n)]** | **Control   [% (events/n)]** | **OR (95%CI)^a^** | **P-value test of same effect across strata** |
| --- | --- | --- | --- | --- | --- |
| **Health centre/outpatient consultations for COVID-19-like illness (extended follow-up)** | | | | | |
| Overall | | 0.78 (125/16009) | 0.91 (143/15764) | 0.84 (0.56-1.25) |  |
| Sex | Male | 0.97 (73/7507) | 0.97 (72/7439) | 0.97 (0.61-1.56) | 0.21 |
|  | Female | 0.61 (52/8502) | 0.85 (71/8325) | 0.70 (0.43-1.13) |  |
| Age | <50 years | 0.75 (107/14354) | 0.88 (124/14041) | 0.82 (0.55-1.21) | 0.62 |
|  | >=50 years | 1.09 (18/1655) | 1.10 (19/1723) | 0.98 (0.43-2.23) |  |
| **All-cause mortality (extended follow-up)** | | | | | |
| Overall | | 0.46 (92/19965) | 0.42 (82/19602) | 1.09 (0.83-1.43) |  |
| Sex | Male | 0.45 (42/9382) | 0.52 (48/9224) | 0.86 (0.58-1.26) | 0.10 |
|  | Female | 0.47 (50/10583) | 0.33 (34/10378) | 1.43 (0.93-2.18) |  |
| Age | <50 years | 0.26 (47/17957) | 0.20 (35/17523) | 1.30 (0.87-1.95) | 0.33 |
|  | >=50 years | 2.24 (45/2008) | 2.26 (47/2079) | 0.97 (0.66-1.44) |  |

a: Estimated in logistic regression models with generalised estimating equation-based correction for cluster. Adjusted for zone and the cluster balancing variables: number of people, proportion aged >50 years and proportion of households with functioning electricity.

**Table D: Effects of mask distribution on reported COVID-19-like illness and health centre consultations stratified by intensity of follow-up.**

|  | **Intervention   [% (events/n)]** | **Control   [% (events/n)]** | **OR (95%CI)^a^** | **P-value test of same effect across strata** |
| --- | --- | --- | --- | --- |
| **Self-reported COVID-19-like illness** | | | | |
| 1 call | 0.74 (96/12900) | 0.93 (123/13157) | 0.79 (0.43-1.46) | 0.92 |
| 2+ calls | 3.82 (148/3879) | 4.90 (165/3370) | 0.82 (0.55-1.23) |  |
| **Health centre/outpatient consultations for COVID-19-like illness** | | | | |
| 1 call | 0.21 (25/12130) | 0.21 (26/12394) | 0.82 (0.37-1.80) | 0.95 |
| 2+ calls | 1.47 (57/3879) | 2.05 (69/3370) | 0.84 (0.52-1.37) |  |
| **Health centre/outpatient consultations for COVID-19-like illness (extended follow-up)** | | | | |
| 1 call | 0.42 (51/12130) | 0.57 (71/12394) | 0.71 (0.36-1.40) | 0.40 |
| 2+ calls | 1.91 (74/3879) | 2.14 (72/3370) | 0.98 (0.63-1.51) |  |
| **Self-reported COVID-19-like illness** | | | | |
| 1 call: Male | 0.98 (59/6022) | 1.07 (66/6150) | 0.89 (0.47-1.66) | 0.74 |
| 1 call: Female | 0.54 (37/6878) | 0.81 (57/7007) | 0.67 (0.33-1.38) |  |
| 2+ calls: Male | 4.02 (73/1815) | 4.82 (77/1598) | 0.90 (0.58-1.40) |  |
| 2+ calls: Female | 3.63 (75/2064) | 4.97 (88/1772) | 0.76 (0.48-1.22) |  |
| **Health centre/outpatient consultations for COVID-19-like illness** | | | | |
| 1 call: Male | 0.32 (18/5692) | 0.27 (16/5841) | 0.95 (0.40-2.24) | 0.47 |
| 1 call: Female | 0.11 (7/6438) | 0.15 (10/6553) | 0.59 (0.22-1.57) |  |
| 2+ calls: Male | 1.65 (30/1815) | 2.25 (36/1598) | 0.86 (0.51-1.45) |  |
| 2+ calls: Female | 1.31 (27/2064) | 1.86 (33/1772) | 0.82 (0.41-1.63) |  |
| **Health centre/outpatient consultations for COVID-19-like illness (extended follow-up)** | | | | |
| 1 call: Male | 0.54 (31/5692) | 0.65 (38/5841) | 0.79 (0.36-1.74) | 0.77 |
| 1 call: Female | 0.31 (20/6438) | 0.50 (33/6553) | 0.60 (0.29-1.25) |  |
| 2+ calls: Male | 2.31 (42/1815) | 2.13 (34/1598) | 1.20 (0.73-1.96) |  |
| 2+ calls: Female | 1.55 (32/2064) | 2.14 (38/1772) | 0.79 (0.44-1.43) |  |

a: Estimated in logistic regression models with generalised estimating equation-based correction for cluster. Adjusted for zone and the cluster balancing variables: number of people, proportion aged >50 years and proportion of households with functioning electricity.

**Table E: Effects of mask distribution on reported COVID-19-like illness and health centre consultations among individuals providing information on their own behalf.**

|  |  | **Intervention   [% (events/n)]** | **Control   [% (events/n)]** | **OR (95%CI)^a^** | **P-value test of same effect across strata** |
| --- | --- | --- | --- | --- | --- |
| **Self-reported COVID-19-like illness until telephone follow-up** | | | | | |
| Overall | | 2.79 (195/6996) | 3.42 (223/6514) | 0.81 (0.58-1.14) |  |
| Sex | Male | 3.30 (107/3244) | 3.95 (117/2962) | 0.83 (0.57-1.22) | 0.83 |
|  | Female | 2.35 (88/3752) | 2.98 (106/3552) | 0.79 (0.53-1.19) |  |
| **Health centre/outpatient consultations for COVID-19-like illness during 4 months** | | | | | |
| Overall | | 0.84 (58/6925) | 1.15 (74/6459) | 0.77 (0.54-1.11) |  |
| Sex | Male | 1.12 (36/3212) | 1.46 (43/2943) | 0.82 (0.51-1.31) | 0.72 |
|  | Female | 0.59 (22/3713) | 0.88 (31/3516) | 0.71 (0.41-1.24) |  |
| **Health centre/outpatient consultations for COVID-19-like illness (extended follow up)** | | | | | |
| Overall | | 1.36 (94/6925) | 1.81 (117/6459) | 0.75 (0.50-1.11) |  |
| Sex | Male | 1.81 (58/3212) | 2.07 (61/2943) | 0.87 (0.53-1.43) | 0.21 |
|  | Female | 0.97 (36/3713) | 1.59 (56/3516) | 0.61 (0.37-0.99) |  |

a: Estimated in logistic regression models with generalised estimating equation-based correction for cluster. Adjusted for zone and the cluster balancing variables: number of people, proportion aged >50 years and proportion of households with functioning electricity.


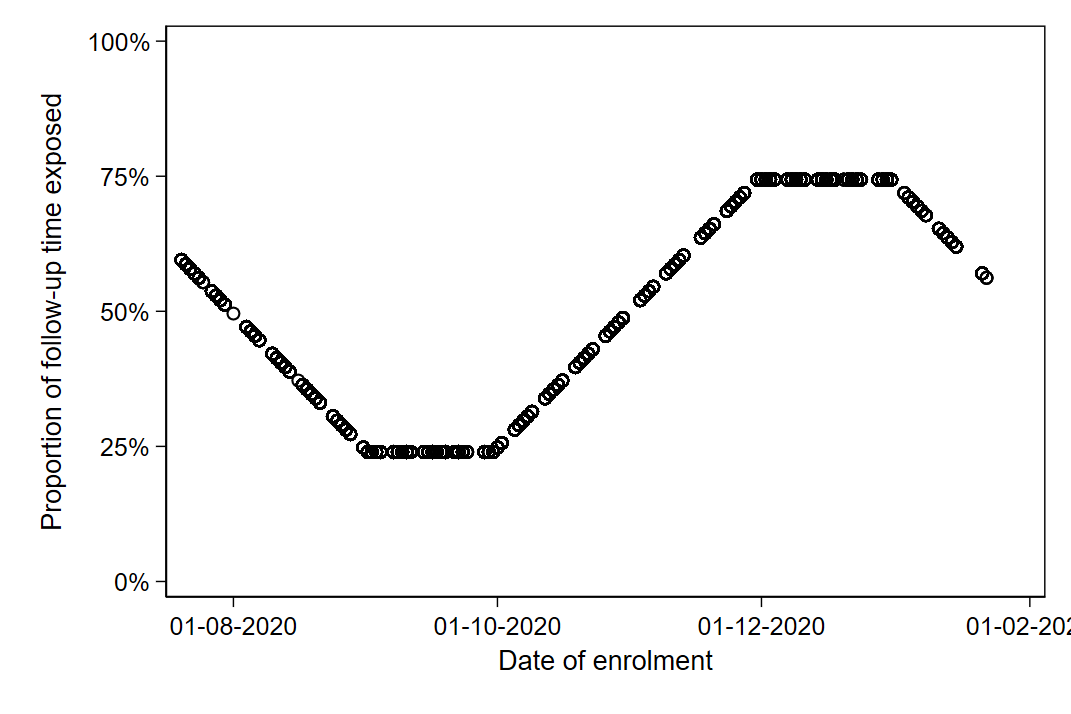


**Fig J: Proportion of the 121 days of follow-up time in higher circulation periods.**

**Table F: Effects of mask distribution on reported COVID-19-like illness, health centre consultations and mortality stratified by proportion of follow-up time during period with higher COVID-19 circulation.**

|  | **Intervention   [% (events/n)]** | **Control   [% (events/n)]** | **OR (95%CI)^a^** | **P-value test of same effect across strata (continuous^b^)** |
| --- | --- | --- | --- | --- |
| **Self-reported COVID-19-like illness** | | | | |
| <=50% | 1.63 (156/9589) | 1.50 (143/9528) | 0.90 (0.60-1.35) | 0.48 (0.20) |
| >50% | 1.22 (88/7190) | 2.07 (145/6999) | 0.70 (0.39-1.25) |  |
| **Health centre/outpatient consultations for COVID-19-like illness** | | | | |
| <=50% | 0.53 (48/9142) | 0.56 (51/9044) | 0.84 (0.50-1.42) | 0.96 (0.25) |
| >50% | 0.50 (34/6867) | 0.65 (44/6720) | 0.87 (0.42-1.77) |  |
| **All-cause mortality** | | | | |
| <=50% | 0.25 (29/11588) | 0.19 (22/11426) | 1.38 (0.82-2.33) | 1.00 (0.27) |
| >50% | 0.27 (23/8377) | 0.18 (15/8176) | 1.38 (0.76-2.52) |  |

a: Estimated in logistic regression models with generalised estimating equation-based correction for cluster. Adjusted for zone and the cluster balancing variables: number of people, proportion aged >50 years and proportion of households with functioning electricity. b: Proportion of time with higher exposure included as a continuous variable.

**Table G: Explorative analyses. Effects of mask distribution on reported COVID-19-like illness, health centre consultations and mortality among participants registered in the area before enrolment and present to receive information at enrolment.**

|  |  | **Intervention   [% (events/n)]** | **Control   [% (events/n)]** | **OR (95%CI)^a^** | **P-value test of same effect across strata** |
| --- | --- | --- | --- | --- | --- |
| **Registered in the Health and Demographic Surveillance system before enrolment** | | | | | |
| **Self-reported COVID-19-like illness** | | | | | |
| Overall | | 1.44 (217/15066) | 1.75 (269/15397) | 0.79 (0.55-1.13) |  |
| Sex | Male | 1.66 (116/7001) | 1.88 (135/7181) | 0.84 (0.57-1.25) | 0.51 |
|  | Female | 1.25 (101/8065) | 1.63 (134/8216) | 0.74 (0.49-1.12) |  |
| **Health centre/outpatient consultations for COVID-19-like illness** | | | | | |
| Overall | | 0.51 (73/14413) | 0.59 (87/14710) | 0.84 (0.56-1.28) |  |
| Sex | Male | 0.63 (42/6718) | 0.67 (46/6912) | 0.92 (0.56-1.51) | 0.58 |
|  | Female | 0.40 (31/7695) | 0.53 (41/7798) | 0.76 (0.42-1.36) |  |
| **All-cause mortality during 4 months of follow-up** | | | | | |
| Overall | | 0.26 (47/17854) | 0.20 (36/18171) | 1.32 (0.88-2.00) |  |
| Sex | Male | 0.25 (21/8350) | 0.24 (20/8500) | 1.06 (0.56-2.03) | 0.66 |
|  | Female | 0.27 (26/9504) | 0.17 (16/9671) | 1.65 (0.93-2.93) |  |
| **Present on date of enrolment** | | | | | |
| **Self-reported COVID-19-like illness** | | | | | |
| Overall | | 1.56 (145/9276) | 1.85 (174/9388) | 0.82 (0.59-1.14) |  |
| Sex | Male | 2.14 (74/3450) | 1.91 (70/3657) | 1.09 (0.72-1.64) | 0.04 |
|  | Female | 1.22 (71/5826) | 1.81 (104/5731) | 0.65 (0.44-0.97) |  |
| **Health centre/outpatient consultations for COVID-19-like illness** | | | | | |
| Overall | | 0.51 (46/8946) | 0.64 (58/9039) | 0.79 (0.50-1.25) |  |
| Sex | Male | 0.66 (22/3333) | 0.74 (26/3523) | 0.88 (0.49-1.56) | 0.64 |
|  | Female | 0.43 (24/5613) | 0.58 (32/5516) | 0.73 (0.40-1.33) |  |
| **All-cause mortality during 4 months of follow-up** | | | | | |
| Overall | | 0.29 (32/11025) | 0.22 (24/11032) | 1.18 (0.69-2.03) |  |
| Sex | Male | 0.36 (15/4152) | 0.30 (13/4295) | 1.06 (0.49-2.30) | 0.66 |
|  | Female | 0.25 (17/6873) | 0.16 (11/6737) | 1.33 (0.64-2.76) |  |

a: Estimated in logistic regression models with generalised estimating equation-based correction for cluster. Adjusted for zone and the cluster balancing variables: number of people, proportion aged >50 years and proportion of households with functioning electricity.

### **Pretrial mortality**

Between January 2013 and December 2019, the 181 clusters had a total of 55,016 persons aged 10+ years under surveillance (median per cluster: 309, IQR: 269-349) and we registered 1842 deaths (median per cluster: 10, IQR: 7-13. The crude mortality in this population was 7.50 per 1000 person years, with little difference between the intervention and control group (Hazard Ratio analysed in a Cox proportional hazards model with age as underlying timescale: 1.03, 95%CI: 0.94-1.13).

# **References**

[1] CEN/TC 79 - Respiratory protective devices [Available from: <https://standards.cencenelec.eu/dyn/www/f?p=CEN:110:0::::FSP_PROJECT,FSP_ORG_ID:32928,6062&cs=177A454543AEF0B8CDF44D4BA501112ED>.

[2] Aydin O, Emon B, Cheng S, Hong L, Chamorro LP, Saif MTA. Performance of fabrics for home-made masks against the spread of COVID-19 through droplets: A quantitative mechanistic study. Extreme Mech Lett. 2020;40:100924.
