# Supplementary material for: Effect of distributing locally produced cloth facemasks on COVID-19-like illness and all-cause mortality–a cluster-randomised controlled trial in urban Guinea-Bissau
Source: PLOS Glob Public Health. 2024 Feb 13;4(2):e0002901. doi: 10.1371/journal.pgph.0002901 (PMC10863890; doi:10.1371/journal.pgph.0002901)
Supplement: S1 Protocol — (PDF) [file pgph.0002901.s002.pdf]

Study Protocol (Translated from the master version in Portuguese)

Title:	Evaluation of Locally Produced Cloth Face Mask on COVID-19 and Respiratory Illnesses Prevention at the Community Level - a Cluster-randomized Trial
Principal Investigator:	Amabelia Rodrigues<br>Epidemiologis, PhD, Projecto Saúde Bandim<br>Contact: a.rodrigues@bandim.org; Tel. +245 966078659
Co-Investigators:	Projecto Saúde Bandim:<br>Justiniano Martins, Else Ca, Cesario Martins<br><br>Southern Danish University (SDU):<br>Ane Fisker, Peter Aaby, Christine Benn, Line Møller Pedersen<br>Kjeld Jensen, Dylan Cawthorne<br><br>Engineers without Borders, Denmark:<br>Dorte Lindegaard Madsen, Ida Højgaard
Institution:	Projecto Saúde Bandim<br>Contact: Carlos Cabral, Administrador<br>c.cabral@bandim.org; Tel. +245 966620077

Índice

INTRODUÇÃO 4

JUSTIFICATION 4

OBJETIVES 4

METHODS 5

- **Study location** 5
- **Study design** 5
- **The intervention** 5
- **HYPOTHESIS** 6

STUDY PROCEDURES 7

- **Randomisation** 7
- **Follow up (all clusters)** 8
- **Sample size calculations** 8
- **Evaluation of compliance** 8

ETHICAL CONSIDERATIONS 9

DISSEMINATION 9

REFERENCES 10

SIGLAS E ABREVIATURAS

CDC	Centro de Controlo de Doenças,
IQR	Intervalo interquartil
OMS	Organização Mundial de Saúde
PSB	Projecto Saúde Bandim

INTRODUÇÃO

The COVID-19 pandemic caused by the new SARS-CoV-2 coronavirus has already caused more than 2.6 million cases and >180,000 deaths worldwide. Despite preventive measures recommended in advance to the public such as hand hygiene, avoiding touching mouth, eye and nose, physical distancing and even radical measures such as blockades in several countries, the number of cases continues to rise. The knowledge to date points to person-to-person transmission as the main route through respiratory droplets and direct contact with infected persons and indirect contact with surfaces in the immediate environment or with objects used by the infected person (https://www.who.int/news-room/commentaries/detail/modes-of-transmission-of-virus-causing-covid-19-implications-for-ipc-precaution-recommendations).

This virus has shown an extremely efficient transmission with an exponential increase in the number of infections and affected sites. In addition, asymptomatic or pre-symptomatic individuals are transmitting the infection.

Asian countries such as China, Taiwan, Hong Kong, South Korea, Singapore and Japan, has always have recommended the use of face masks as part of the public's measures, and it has been widely used against COVID-19. The WHO and most countries in Europe and America have recommended the use of face masks only for healthcare personnel or individuals with symptoms or those caring for infected people, but this has changed recently, with the CDC now recommending face masks for everyone (https://www.cdc.gov/coronavirus/2019-ncov/prevent-getting-sick/cloth-face-cover.html), and the WHO stating that it may be a good idea (https://www.youtube.com/watch?v=2d8x1njGbFE).

However, all agree that there is a lack of good data on the community impact of face masks, particularly on the use of cloth face masks (https://www.bmj.com/content/369/bmj.m1435).

Previous studies were mostly conducted in situations with little perceived threat and showed low compliance. Furthermore, masks can be of different qualities and very little is known about the utility of cloth face masks at community level (https://www.bmj.com/content/369/bmj.m1435).

No studies have been done in an African context.

JUSTIFICATION

All over the world it has been recommended to use face masks made by the population itself as a way to help protecting oneself and others due to the scarcity of appropriate masks. In Guinea-Bissau, wearing a mask in public is now compulsory. However, different models are improvised and the real value in prevention is unknown. Thus, there is a need to evaluate the effect of this protection when performed with an improved and tested mask on the occurrence of infection and its severity.

OBJETIVES

We aim to conduct a randomised controlled trial to assess the impact of providing cloth face masks made to a tested standard compared to non-standard masks in an urban population during the course of a pandemic.

METHODS

Study location

Bandim Health Project (BHP) study area in Bissau. The study area comprises the households of approximately 100,000 individuals, among whom 73,500 are older than 10 years. It covers 6 bairros and is divided into 324 sub-zones. Each sub-zone has a median of 232 individuals (IQR 194-261). Persons in three of these neighbourhoods with 182 sub-areas will be eligible for the study (~43000 persons).

Study design

A two-arm randomised intervention trial, with the unit of randomisation being the sub-zones within the neighbourhood. The arms will be:

- Intervention: Sub-zones allocated to receive enhanced face masks to a tested standard, which will be distributed to all residents aged 10 years and over, plus information on preventive measures for COVID-19.
- Control: Sub-areas allocated to receive information on preventive measures for COVID-19.

The intervention

<u>Cloth face masks:</u>

Face masks will be sewn with locally available cotton cloth, elastic bands and metal wire, following a recipe developed by engineers at the Technical Faculty of the University of Southern Denmark, and tested in a laboratory that CE certifies masks for hospital use (See Figure 1).

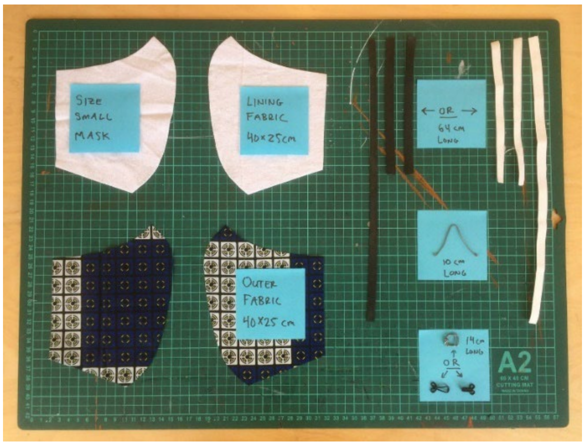

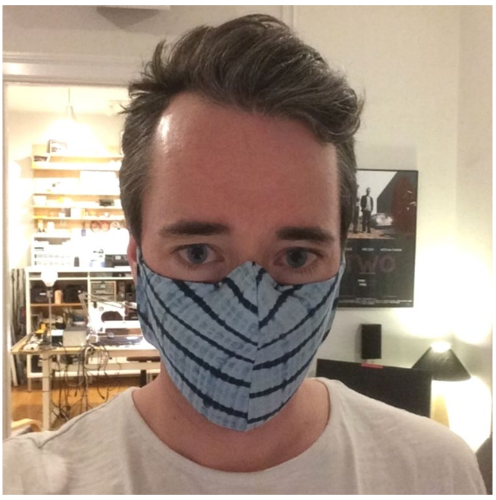

The cloth masks were tested by "FORCE" (https://forcetechnology.com/en/services/test-approval-personal-protective-equipment-ppe) according to the European standard (EN 149 RfU), and the result shows that they have a filtering capacity of about 11 to 19%, depending on the size of the particles. The results do not provide a specific answer to the filtering capacity in relation to aerosols from a sneeze with the corona virus. The corona aerosol droplets are about 3 micrometers and thus 10 times larger than the particles to which the cloth masks were exposed in the FORCE test.

In the intervention households, two masks for each person will be distributed to all residents over the age of 10 and they will be instructed to wear them whenever they go out or when they are in contact with many individuals who are not from the household. They will also be instructed to wear them at all times if there is a sick person in the home.

They will be instructed to wash the mask with soap before use, every day each time it is used and not to touch them constantly (see pictogram).

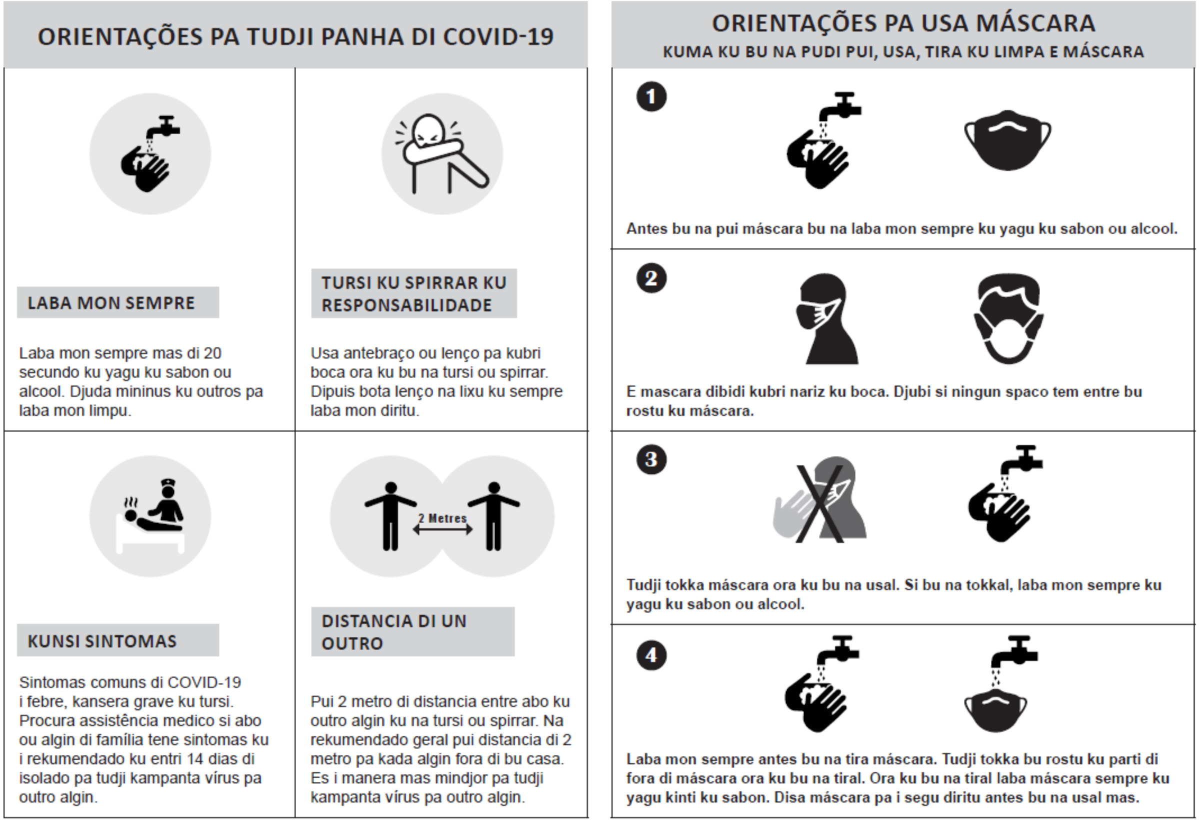

<u>Information on preventive measures for COVID-19:</u> Key messages given in-country for the prevention of COVID-19 will be provided. These include washing hands frequently with soap or using alcohol gel solution or bleach solution, physical distancing, avoiding crowds, keeping safe distance (1 m distance here), avoiding shaking hands and kissing, etc. The overall measures also include staying indoors and moving around only during the permitted period and for essential activities. Messages will be short and with pictures (see pictogram).

Hypothesis

The use of the homemade face masks and their disposal, along with preventive messages, will reduce reported COVID-like illness by 8% and all-cause mortality by 20% in families that received the face masks.

ENDPOINTS

COVID-like illness: defined as self-reported major symptoms of COVID-19 (three or more of the following: fever, cough, fatigue, shortness of breath, loss of smell/taste) or/and reported positive test (done to them by health staff);

- Serious illness: as above including hospitalisation
- Death: any death during the follow-up period

The proportion of individuals with a positive rapid Ig G test can be considered as a secondary end point if testing is available in the country.

STUDY PROCEDURES

Randomisation

Prior to the start of the study, we will classify all subzones according to target population size, age distribution (proportion of individuals older than 50 years) and socioeconomic status (proportion of households with functional electricity during the past 2 years). To ensure balanced groups with respect to these factors, we will use restricted randomisation. Sub-zones will be randomised prior to the start of the study using a computer algorithm to draw a randomised combination of the sub-areas within each neighbourhood that is checked for co-variable balance. The trial is not blinded.

All clusters

The study households will be visited during the period when people can move around (between 7am and 2pm). The PSB field assistant will use personal protection (face mask, disinfectant and physical distance in the field).

The assistant will bring the latest census lists and note if there are any new arrivals>10 years old or anyone who has died/changed. They will be asked how many people currently live in the household. A list of all residents aged 10+ from the PSB database will be used to check and update any unregistered residents.

Up to three telephone numbers for household members will be collected.

Baseline household information such as household characteristics (roof, cemented porch, education of household head, source of water supply) are already available on the registers.

Summary of data to be collected at baseline:

- Date of visit
- Demographics and telephone numbers will be checked and updated if necessary
- Present and reason for absence with date if absent or dead
- Who sleeps in the same room and bed with whom, type of ceiling (known risk factors for infection).

All clusters will receive information about covid-19 verbally and written inside a plastic disinfected with 70% alcohol.

Specifically for intervention clusters:

Two cloth face masks will be provided for each person > 10 years of age, and will be provided, along with additional information on how to handle the face mask.

The following additional information will be collected:

- Who has received masks; If someone is temporarily absent, two masks would be left for them.
- Availability/intention to use the mask or any reason for not using it

Follow up (all clusters)
The follow-up will last four (4) months. Every 6-8 weeks, a fieldworker will call a person or a close relative in the household asking for information about the illness in the last 6-8 weeks, as indicated in endpoints or survival. The interviewee will be noted. At the end of the follow-up period, a physical visit will be made if no response can be obtained from a participant. Cloth face masks will be distributed to the control group at the end of the study.

Data to be collected at follow-up:

- Self-reported illness, symptoms, diagnosis, COVID-19 (yes/no, dates).

- Consultation, hospitalisation, death (yes/no, dates, location)

- Absence and reason - where travelled to, moving, etc.

- Attendance at events with many people (crying, funeral, etc.)

- Someone assisted with COVID-19

- Use of masks: whether they are used when the person leaves home or if there is a sick person at home

Other preventive measures and if not, the reasons will be collected

For reported deaths interviews will be conducted on the causes and circumstances of death for verbal autopsy using a standard questionnaire adapted from WHO (attached).

Sample size calculations
With the expected 10% refusal to participate, we would have 19500 in each randomisation group. We used a logistic regression with generalised estimating correction (GEE) based on effect by clusters. We ran 10,000 simulations with a baseline mortality risk of 1%, allowing a range between 0.5% and 1.5% for individual subareas and using a uniform mortality distribution. Based on the estimated number of enrolments in each subzone and assuming the true mortality reduction by the intervention is 21%, we will have 53% power to demonstrate an effect of the intervention. For more frequent outcomes we will have greater power. For example, for disease (predicted baseline rate of 10%, uniform range 5-15%) we will have 58% power to show a reduction if the true effect is an 8% reduction and 80% power to show a reduction if the true effect is a 10% reduction.

Evaluation of compliance
Real life observation of how many people are wearing a face mask when they leave home and return in both intervention and control areas will be done through spot checks.

Around 5% of selected intervention and control areas will be randomly selected and remote observation will be carried out in the morning from 8 am to 2 pm to record the number of people leaving home and wearing a face mask.

ETHICAL CONSIDERATIONS

INASA and COES have already approved the project. Approval from the National Health Ethics Committee will be sought before commencement. The heads of household will be asked for oral consent after the explanation of the study. The intervention is not related to any serious risk. General information about COVID-19, namely about the importance of applying safety precautions and that wearing a mask is only an addition to the other measures.

All contacts of the BHP with the households will be restricted to the minimum necessary and the recommended protective measures and physical distance will be followed precisely by field workers and household residents during indispensable contacts within the scope of the study.

DISSEMINATION

The study report will be made available to the Health Emergency Operations Centre for possible use of the results and deposited in the library of the National Institute of Public Health. The data will be published in a scientific journal and may be used for postgraduate work. The results of the intervention will be widely disseminated to health personnel at all levels.

REFERENCES

Mass masking in the COVID-19 epidemic: people need guidance.

Leung CC, Lam TH, Cheng KK.Lancet. 2020 Mar 21;395(10228):945. doi: 10.1016/S0140-6736(20)30520-1. Epub 2020 Mar 3.

COVID-19: Face masks and human-to-human transmission.

Liu X, Zhang S.Influenza Other Respir Viruses. 2020 Mar 29. doi: 10.1111/irv.12740. Online ahead of print.

Rational use of face masks in the COVID-19 pandemic.

Feng S, Shen C, Xia N, Song W, Fan M, Cowling BJ.Lancet Respir Med. 2020 Mar 20. doi: 10.1016/S2213-2600(20)30134-X. Online ahead of print.

Epidemiology, causes, clinical manifestation and diagnosis, prevention and control of coronavirus disease (COVID-19) during the early outbreak period: a scoping review.

Adhikari SP, Meng S, Wu YJ, Mao YP, Ye RX, Wang QZ, Sun C, Sylvia S, Rozelle S, Raat H, Zhou H.Infect Dis Poverty. 2020 Mar 17;9(1):29. doi: 10.1186/s40249-020-00646-x.

Knowledge, attitudes, and practices towards COVID-19 among Chinese residents during the rapid rise period of the COVID-19 outbreak: a quick online cross-sectional survey.

Zhong BL, Luo W, Li HM, Zhang QQ, Liu XG, Li WT, Li Y.Int J Biol Sci. 2020 Mar 15;16(10):1745-1752. doi: 10.7150/ijbs.45221. eCollection 2020.

COVID-19 transmission through asymptomatic carriers is a challenge to containment.

Yu X, Yang R.Influenza Other Respir Viruses. 2020 Apr 4. doi: 10.1111/irv.12743. Online ahead of print.
