# Supplementary material for: Effect of distributing locally produced cloth facemasks on COVID-19-like illness and all-cause mortality–a cluster-randomised controlled trial in urban Guinea-Bissau
Source: PLOS Glob Public Health. 2024 Feb 13;4(2):e0002901. doi: 10.1371/journal.pgph.0002901 (PMC10863890; doi:10.1371/journal.pgph.0002901)
Supplement: Attachment — Submitted filename: Response.docx [file pgph.0002901.s003.docx]

1. We note your Data Availability statement: "Data sharing can take place after ethics approval. Requests to corresponding author."

PLOS journals require authors to make all data necessary to replicate their study’s findings publicly available without restriction at the time of publication. When specific legal or ethical restrictions prohibit public sharing of a data set, authors must indicate how others may obtain access to the data.

Please address the following:

a) At this time disclose the reason for this data restriction and provide a point of contact, preferably an email, for the ethical body mandating the restriction of this data. Please note that this point of contact cannot be an author of this study.

b) If there are no restrictions on this dataset, please provide your data by uploading it as a Supporting Information file or depositing it in a stable repository.

We hope to hear from you soon.

***Response:*** *We have expanded the explanation and have now written:*

*“As part of the data transfer agreement between the National Institute of Public Health (INASA) and the Bandim Health Project, access to data will granted when covered by an ethical permission and approved the data access committee consisting of the director of INASA, the steering board of the Bandim Health Project and key researchers involved in the study. The Guinean Ethics Committee (Comité Nacional de Ética em Pesquisa na Saúde) can be contacted at* [*nabangna.julho2009@gmail.com*](mailto:nabangna.julho2009@gmail.com) *or* [*djicoblama@gmail.com*](mailto:djicoblama@gmail.com)*.”*

2. Some material included in your submission may be copyrighted. According to PLOS’s copyright policy, authors who use figures or other material (e.g., graphics, clipart, maps) from another author or copyright holder must demonstrate or obtain permission to publish this material under the Creative Commons Attribution 4.0 International (CC BY 4.0) License used by PLOS journals. Please closely review the details of PLOS’s copyright requirements here: PLOS Licenses and Copyright. If you need to request permissions from a copyright holder, you may use PLOS's Copyright Content Permission form.

Please respond directly to this email or email the journal office and provide any known details concerning your material's license terms and permissions required for reuse, even if you have not yet obtained copyright permissions or are unsure of your material's copyright compatibility.

Potential Copyright Issues:

S2 Figure: Please confirm (a) that you are the photographer; or (b) provide written permission from the photographer to publish the photo(s) under our CC-BY 4.0 license.

***Response:*** *Co-author Dylan Cawthorne was the photographer and has provided the photograph for the paper. The information has been added.*

S3 Figure and S4 Figure: Please confirm whether you drew the images / clip-art within the figure panels by hand. If you did not draw the images, please provide (a) a link to the source of the images or icons and their license / terms of use; or (b) written permission from the copyright holder to publish the images or icons under our CC-BY 4.0 license. Alternatively, you may replace the images with open source alternatives. See these open source resources you may use to replace images / clip-art:

<https://commons.wikimedia.org/>

<https://openclipart.org>

***Response:***

*S3 Figure was created by our collaborator at Engineers without Boarders (Ida Højgaard). A copyright form has been uploaded.*

*S4 figure has been redrawn using open-source icons and a mask icon drawn by co-author Dylan Cawthorne. The information has been added to the figure.*

S5 Figure includes an image of an identifiable person. Please provide written confirmation or release forms, signed by the subject(s) (or their parent/legally authorized guardian), giving permission to be photographed and to have their images published under our CC-BY 4.0 license.

Otherwise, we kindly request that you remove the photograph.

***Response:*** *Photograph Release forms from all individuals have been collected and uploaded.*

We've returned your manuscript to your account. Please resolve these issues and resubmit your manuscript within 30 days. If you need more time, please email the journal office at globalpubhealth@plos.org. We are happy to grant extensions of up to one month past this due date. If we do not hear from you within 30 days, we will withdraw your manuscript on your behalf.

Please log on to PLOS Editorial Manager at https://eur03.safelinks.protection.outlook.com/?url=https%3A%2F%2Fwww.editorialmanager.com%2Fpgph%2F&data=05%7C01%7Cafisker%40health.sdu.dk%7C17ddcd6b273a4eb45ec908dbb8593bf6%7C9a97c27db83e4694b35354bdbf18ab5b%7C0%7C0%7C638306465644849293%7CUnknown%7CTWFpbGZsb3d8eyJWIjoiMC4wLjAwMDAiLCJQIjoiV2luMzIiLCJBTiI6Ik1haWwiLCJXVCI6Mn0%3D%7C3000%7C%7C%7C&sdata=tOgIUPDDYk8b%2FNfQA1DyO2k40v3kQuGqLHidpy8lMMo%3D&reserved=0 to access your manuscript. You will find your manuscript in the 'Submissions Sent Back to Author' link under the New Submissions menu. Be sure to remove your previous manuscript file if you are uploading a new file in response to these requests. After you've made the changes requested above, please be sure to view and approve the revised PDF after rebuilding the PDF to complete the resubmission process.

We are requesting these changes to comply with the PLOS Global Public Health submission guidelines (https://eur03.safelinks.protection.outlook.com/?url=https%3A%2F%2Fjournals.plos.org%2Fglobalpublichealth%2Fs%2Fsubmission-guidelines&data=05%7C01%7Cafisker%40health.sdu.dk%7C17ddcd6b273a4eb45ec908dbb8593bf6%7C9a97c27db83e4694b35354bdbf18ab5b%7C0%7C0%7C638306465644849293%7CUnknown%7CTWFpbGZsb3d8eyJWIjoiMC4wLjAwMDAiLCJQIjoiV2luMzIiLCJBTiI6Ik1haWwiLCJXVCI6Mn0%3D%7C3000%7C%7C%7C&sdata=OHKP744WfS2Y5sVwMVehrMUCkTMEBDVGXfo%2FwONGwnI%3D&reserved=0). Please note that we won't send your manuscript for review until you have resolved the above requests.

***Response:*** *Thank you for the opportunity to submit a revised manuscript which is hopefully now fully compliant with the guidelines. I am however a little confused about the process. You write here that you will not send our paper for review before receiving our corrections, but our paper was sent for review some months ago. The process looks like this:*

*May 19:	We submitted the paper ref. PGPH-D-23-00950*

*May 23:	Edits were requested by your colleague Jonathan Binamira Due, revised version submitted.*

*30 June:	We received information that the editor had become unavailable and that another editor	would be found.*

*4 August:	We again received information that the editor had become unavailable and that another	editor would be found.*

*23 August:	We received reviewer comments and submitted a revised manuscript taking these into	account on 1 September (this manuscript became PGPH-D-23-00950R1*

*Sept 6:	Your colleague Shaira Joy Petancio requested edits on PGPH-D-23-00950R1 these were	submitted on Sept 8.*

*Sept 18:	You requested edits on PGPH-D-23-00950R1*

*An many of these steps appear double, I wanted to make sure that we do not have some duplication of the manuscript I the system.*
