# Supplementary material for: Effect of distributing locally produced cloth facemasks on COVID-19-like illness and all-cause mortality–a cluster-randomised controlled trial in urban Guinea-Bissau
Source: PLOS Glob Public Health. 2024 Feb 13;4(2):e0002901. doi: 10.1371/journal.pgph.0002901 (PMC10863890; doi:10.1371/journal.pgph.0002901)
Supplement: Attachment — Submitted filename: 20231218_Response.docx [file pgph.0002901.s004.docx]

Below follows a point-to-point response to the comments raised by the editorial team and the reviewer.

Journal Requirements:

1. Please review your reference list to ensure that it is complete and correct. If you have cited papers that have been retracted, please include the rationale for doing so in the manuscript text, or remove these references and replace them with relevant current references. Any changes to the reference list should be mentioned in the rebuttal letter that accompanies your revised manuscript. If you need to cite a retracted article, indicate the article’s retracted status in the References list and also include a citation and full reference for the retraction notice.

*Response: We have reviewed the list and believe it to be correct. None of the cited papers have been retracted.*

2. We have noticed that you have uploaded Supporting Information files, but you have not included a list 3of legends. Please add a full list of legends for your Supporting Information files after the references list.
*Response: We have added a legend for S1 Appendix: “S1 Appendix (Supplementary Methods, supplementary Results, S1-S10 Figures, S1-S7 Tables)”*

Reviewer #2: The authors report the findings of a cluster-randomized trial in which cloth facemasks were distributed and telephone interviews were conducted to assess the impact of COVID-19-like illnesses and mortality over 4 months. The authors should be commended for carrying out such a large-scale trial during the pandemic, using locally-sourced materials to produce face masks. The trial was conducted within the Health and Demographic Surveillance System in Guinea-Bissau, providing a well-enumerated population. The authors performed appropriate statistical tests of their data from which to draw conclusions. Unfortunately, the intervention did not impact the study outcomes. The authors acknowledge several limitations, including lack of impact on behaviors, spillover effects of the intervention, and limited recall. In general, the manuscript is well-written and highlights the numerous challenges of conducting studies during outbreaks.

*Response: Thank you for these comments and for your constructive suggestions.*

Major comments:
Lines 105-107: Were all individuals within a household enrolled? Or was one individual selected per household? Please provide additional details around enrollment selection. This is important for the interpretation of the findings.

*Response: We have clarified that all household members were eligible for enrolment, and that other family members could accept on their behalf (lines 108-9).*

Line 145-146: Clarify whether “positive test” refers to PCR, antigen, and/or antibody testing.

*Response: Only PCR tests were available in the country. We have added the information to the description of the number of cases (lines 90-91) and further specified “PCR-tests” in line 146.*

Lines 157-159: How was the observer able to distinguish study facemasks? Were they clearly distinctive from other facemasks? The photos in the supplement do not show obvious differences.

*Response: Based on our experience, it was possible to distinguish them. We have added the information to the supplementary material which now reads: “The study cloth facemasks were recognisable as they had two elastic band extending across the back of the head and the neck, and covered the entire jaw, including the area between the jaw-angle and the ear (S5 Figure)”*

Lines 344-348: A major limitation of the Danish study was the lack of assessment of compliance with face mask use in the intervention arm. This should be highlighted as an important difference from the Bangladeshi study. Face masks can only be expected to have an effect when they are worn, which is relevant to the current study’s findings.
*Response: We have added the information: “Self-reported mask-use (predominantly or fully as recommended) in the intervention group was 93%, but there was no observation of actual use[28].” (lines 350-2).*

Given that the study was mired by logistical constraints of doing research during a pandemic, it would be interesting to get the authors’ perspective on how the scientific community could be better situated to generate evidence during future outbreaks, including a brief mention of lessons learned.
*Response: We think that both the scientific community and public health officials could have learned much more during the pandemic. From our perspective, there were many unused opportunities to learn about the interventions employed to mitigate the impact of COVID-19. As an example, interventions which were short in supply (e.g., vaccines) could have been rolled out in in step-wedged cluster randomized designs. In countries with weaker health information system, HDSS systems such as the Bandim HDSS would be a great platform to assess effects both of vaccines and other mitigating strategies. However, we do not feel that including this discussion in the reporting of the trial outcomes is appropriate. We fear that it would complicate the presentation further and have therefore refrained from doing so. Nevertheless, to emphasize that the HDSS sites could provide important information on the toll of the pandemic, we have updated our statement on mortality patterns and added references to studies which have recently been published from other HDSS sites. The revised text reads: “There is still very limited empirical data on overall mortality during the pandemic based on community data from Africa. Though our trial covers only a narrow time window, it suggests that the impact of COVID-19 on the overall mortality may have been less severe than feared. Hence, as also shown in other African HDSS sites[33, 34], the lower death toll in some African settings cannot be fully explained by lack of data.” (line 393-7)*

Minor comments:
Lines 94-95: It would be helpful to understand more about the mask mandate. Was there any enforcement? When did the mandate end? Was it anticipated to have any effect on the trial results (e.g. improving mask wearing in both the control and intervention groups)?

*Response: The mask mandates continued throughout 2021 and thus beyond the duration of the trial. We have added references to online news articles reporting on the police control of mask use (line 96: “with the police intermittently enforcing the use on streets and in market areas[19,20]”). However, the police checks were not continuous throughout 2020 and 2021.*

*We have clarified in the discussion that the mask-mandate likely increased the use in both groups and the revised discussion reads: “Furthermore, in contrast to when we planned the trial, facemask use was mandated in Guinea-Bissau throughout the trial period. Thus, the information on use obtained through the telephone follow-up is likely influenced by social desirability bias. Nevertheless, the facemask mandates likely increased the mask-use in both the intervention and control group and may have meant that we have been comparing an intervention group who (at times) used the provided cloth facemasks, with a control group who were more likely to use medical facemasks.” (line 329 ff).*

Lines 95-96: It would be helpful to better understand the curfews that were in place. Did they last for the duration of the study? What were the restrictions? If residents could not leave their houses, masks would not be expected to have an effect. However, this does not seem to be the case since many participants reported interacting with groups of >20 people on a regular basis.

*Response: The curfews ceased shortly before we started the trial, this has been clarified (line 97).*

Lines 136-138: It would be helpful to have additional details in the supplement about the tests that were performed to determine the filtration efficiency of the masks. EN DIN 149 is mentioned as the standard, but no citation is provided. For example, what was the flow rate? Was air flow resistance measured? Were measurements assessed after washing and drying the facemasks as this can significantly affect the fabric performance? Were multiple samples of plain-weave cotton fabric tested? Filtration results can vary greatly by thread count. How was this controlled for across production? How were sizes determined for study participants? Were there any acceptability studies?

*Response: Thank you for your suggestions. In the revised version we have written: “A sample of the cloth facemasks produced by investigators using washed plain weaved cotton fabric bought in Guinea-Bissau, were tested by the company FORCE Technology according to EN 149 for medical facemasks[1]. The tested flow rates were 30 and 150 l/min to simulate in- and exhalation, and the particles tested were paraffin oil (0.6 µm) and NaCl (0.4µm). Thus, the filtration ability was tested for sizes substantially smaller than the droplet sizes in e.g. a sneeze (3µm)[2]. Using this standard, FFP2 masks should filter at least 94% of the particles. The results indicated an 11-19% filtration rate dependent on particle size. Airflow resistance was not measured. Testing was not repeated for the masks which were subsequently produced, and acceptability was not investigated.*

*Intervention group participants were provided with masks in size small, medium, large or extra-large dependent on the field worker’s visual assessment of suitable size.”*

Lines 150-151: Soliciting information from family members and neighbors when participants were absent may have resulted in recall bias, though presumably this should have been similar between the control and intervention groups.

*Response: We agree. We therefore performed a sensitivity analysis (S5 Table). We discuss this in line 335ff: “Furthermore, a poorer recall on behalf of other household members (~5 times lower proportion), was also evident. Nevertheless, as the conclusions did not change when restricting the analyses to information being provided by the participant or adjusting for recall period, this should not affect the relative comparison.”*

Table 1: For number of people sleeping in the same room, how can the p-value be 0.001 if the median and IQR were the same in the control and intervention arms?

*Response: We were also puzzled by this and have double-checked. However, with 18,000 per group, smaller departures beyond the central half of the sample, can lead to distribution which are statistical significantly different, though they are small.*

Recall seems to be an issue given the much higher reporting among those who were contacted at 2-month intervals compared with study end only (Lines 287-290 and 293-295).

*Response: We agree. We discuss this on page 17ff: “Our analyses showed higher proportions of events when called with a shorter recall period, hence the recall periods were clearly too long to provide a full picture. (…). Nevertheless, as the conclusions did not change when restricting the analyses to information being provided by the participant or adjusting for recall period, this should not affect the relative comparison.”*

Line 299: Follow-up is duplicated.

*Response: Thank you. Deleted*

While revising your submission, please upload your figure files to the Preflight Analysis and Conversion Engine (PACE) digital diagnostic tool, https://pacev2.apexcovantage.com/. PACE helps ensure that figures meet PLOS requirements. To use PACE, you must first register as a user. Registration is free. Then, login and navigate to the UPLOAD tab, where you will find detailed instructions on how to use the tool. If you encounter any issues or have any questions when using PACE, please email PLOS at figures@plos.org. Please note that Supporting Information files do not need this step.

*Response: All files appear to meet the requirements, but we have nevertheless uploaded the versions which we have downloaded from PACE.*
